# Spatial and temporal distribution characteristics and influencing factors of tourism eco-efficiency in the Yellow River Basin based on the geographical and temporal weighted regression model

**DOI:** 10.1371/journal.pone.0295186

**Published:** 2024-02-20

**Authors:** Donghui Peng, Zongzheng Liang, Yapeng Ding, Liuke Liang, Aohui Zhai, Yan Zhang, Xu Gong

**Affiliations:** 1 Key Research Institute of Yellow River Civilization and Sustainable Development & Collaborative Innovation Center of Yellow River Civilization Provincial Co-Construction, Henan University, Kaifeng, Henan, China; 2 Academy of Regional and Global Governance, Beijing Foreign Studies University, Beijing, China; 3 College of Geography and Environmental Science, Henan University, Kaifeng, Henan, China; 4 Collaborative Innovation Center of Smart Tourism of Henan Province/College of Land and Tourism, Luoyang Normal University, Henan Luoyang, China; 5 Tourism Academy, Henan Normal University, Xinxiang, Henan, China; East China Normal University, CHINA

## Abstract

With economic progression in China, Yellow River Basin serves as a critical economic belt, which has also been recognized as a cradle of Chinese culture. A watershed is a complex structure of social, economic, and natural factors, and the diversity of its components determines its complexity. Studies on the spatiotemporal distribution characteristics and factors influencing the tourism eco-efficiency at the watershed scale are crucial for the sustainable regional socio-economic development, maintaining a virtuous cycle of various ecosystems, and comprehensively considering the utilization and coordinated development of various elements. Based on tourism eco-efficiency, the coordination degree of regional human–land system and the sustainable development levels can be accurately measured. With the tourism eco-efficiency in the Yellow River Basin from 2009 to 2019, the present study considers 63 cities in the Yellow River Basin as the research area by adopting the super-efficiency slacks-based measure (Super-SBM) model. Methods such as trend surface analysis, spatial autocorrelation analysis, elliptic standard deviation analysis, and hot spot analysis were used to explore their spatiotemporal distribution and evolution characteristics. The geographical and temporal weighted regression (GTWR) model was used to determine the factors influencing the tourism eco-efficiency value. The findings are as follows: ①The level of tourism eco-efficiency in the Yellow River Basin is not high, exhibiting a fluctuating upward trend. ②The tourism eco-efficiency in the Yellow River Basin shows significant spatial interdependence and agglomeration. Furthermore, the track of the center of gravity moves from northeast to southwest. ③ The tourism eco-efficiency in the Yellow River Basin is affected by various factors, with the economic development level having the greatest influence.

## Introduction

As the second largest river in China, the Yellow River is regarded as the birthplace of Chinese civilization and is widely referred to as “Mother River.” The Yellow River Basin belongs to one of the earliest developed regions in not only China but also the whole world. Historically, the basin has nurtured Chinese culture. The Yellow River Basin is topographically complicated and climatologically diverse. Typically, the basin displays semi-arid and arid zones, as well as eco-environmental fragility, particularly in the Loess Plateau area, which faces the problems of serious soil erosion and noticeable imbalance of ecosystem [1, 2]. Being densely populated, the Yellow River Basin represents approximately 23.31% of the overall population in China. Nonetheless, due to the developmental difficulties encountered by the region, its economic development level is low [3]. Since 2019, the ecological conservation in the Yellow River Basin and its high-quality development have been identified as the major national strategy, which is favorable for both the economic and social development in the region. Achieving coordinated development of economic growth and environmental protection in the Yellow River Basin has already been a critical topic of research.

Tourism, as an emerging industry, can effectively drive the development of relevant industries with its strong relevance and has become a pillar of economic development in China. Moreover, tourism provides a critical reference for the all-round social and economic development of the Yellow River Basin. With mass tourism, the tourism industry continues to grow. In 2019, 56.49% of the entire tourists in China were visitors from the tourism industry of the Yellow River Basin, and the total tourism income of the region accounted for 51.43% of China’s overall tourism income. Nevertheless, the rapid progress of tourism at a large scale has contributed to air pollution [4], water pollution [5], vegetation destruction [6, 7], and ecological environmental problems. Fossil energy sources consumed and greenhouse gases emitted by tourism activities are the critical driving force of global climate change [8]. To realize the green development of tourism in the Yellow River Basin, it is essential to scientifically understand the economic benefits and ecological environment problems brought by tourism development.

Tourism eco-efficiency can effectively measure the coordination degree of the regional human–land system and the level of sustainable development, and it is a key indicator of the green development of tourism[9]. Moreover, it has been research hot spot for tourism geographers, who have focused mainly on the following aspects: ①Concept analysis. Tourism eco-efficiency has been extensively adopted for evaluating the degree of coordination between economic efficiency and ecological environment [10]. It is an extension of eco-efficiency and is adopted for achieving “maximum economic output with minimum resource consumption and environmental cost” [11]. Gössling introduced tourism eco-efficiency into regional tourism research [12]. However, tourism eco-efficiency is yet to be investigated conclusively. According to Yao and Tian [13], tourism eco-efficiency implies an increase in the expected output in tourism activities and decreases in the energy consumption and carbon emissions to achieve tourism economic benefits. From the industry perspective, Jia opined that the ratio of the commodity value provided by the tourism industry to the amount of environment consumed in a region within a specific time could be used as a measure of tourism eco-efficiency [14]. Although different scholars have different conceptions of tourism eco-efficiency, they are all based on the benefits generated by tourism activities and their impact on the environment. ②Construction of evaluation index system and selection of measurement method. Scholars have mainly used the single ratio method [12, 15, 16], index system method [17], and model construction. Its measurement is based on the input of constructing human, capital and tourism resources, the expected output of society, economy and ecology, and the unexpected output of environmental cost. Among these methods, model construction is favored because of its ability to comprehensively calculate tourism eco-efficiency, with the most widely models being the DEA and SBM [18, 19]. Conventional DEA methods include the reciprocal conversion method [20], directional distance function method [21], and three-stage DEA [22, 23]. However, this method does not consider residuals and cannot effectively solve the input–output relaxation variables problem. The SBM model [24], especially the improved SBM model, such as Super-SBM [25, 26], Super-EBM [27], SBM + Malmquist index [28], SBM-undesirable [29], Malmquist-Luenberger index [30], and other models, helps overcome this problem and better reflect the real situation in evaluating tourism eco-efficiency. ③Spatial and temporal patterns. Jia Liu, Fei Lu, Zheng Hong, and Ziying Wang, studied 30 provinces in China [29, 31], the western region [32], the Yangtze River Delta urban agglomeration [2], and other regions, to investigate the spatiotemporal distribution and influential factors of tourism eco-efficiency. The research content is mostly related to the spatial spillover effect [33], threshold effect [34], spatial correlation characteristics [25], and economic resilience [35]. The spatial and temporal differences of tourism eco-efficiency in different regions are large, and the spatial club convergence phenomenon of "high agglomeration, low agglomeration" is present in space; thus, the efficiency value fluctuates and increases over time. ④Influencing factors and mechanism. The interaction between natural and human factors leads to the diversity and complexity of the spatial and temporal distribution of tourism eco-efficiency. Scholars have studied the influencing factors of tourism eco-efficiency mainly from the spatial dimension. It mainly uses spatial Dubin model [26], Tobit regression model [22], geographical detector [36], social network analysis [37] and other methods. Tourism market, tourism economic scale, tourism industry structure, science and technology level, and government policies have been identified as the main influencing factors; however, the identification of influencing factors in the temporal dimension still needs to be strengthened.

To sum up, the research methods of tourism eco-efficiency are highly diverse, and the research content mainly focuses on the measurement of tourism eco-efficiency at the national and provincial scales and discusses the reasons for the change of tourism eco-efficiency from the spatial dimension. However, there are still some shortcomings in the previous studies. First, watershed is a complex of social, economic, and natural factors, and the diversity of its components determines its complexity. Presently, studies on tourism eco-efficiency at river basin scale, especially in the Yellow River basin, are scarce. Second, the empirical analysis of tourism eco-efficiency mostly focuses on the spatial differentiation characteristics from a static perspective, and the lack of spatial dynamic evolution characteristics of tourism eco-efficiency may lead to biased conclusions. Finally, it is necessary to pay attention to the influence of various factors on tourism eco-efficiency in different periods. Given the complex relationship between humans and land in the Yellow River Basin, the study on the spatial and temporal distribution characteristics and influencing factors of tourism eco-efficiency is beneficial for the sustainable social and economic development of the region, maintenance of the virtuous cycle of various ecosystems, and comprehensive consideration of the utilization and coordinated development of various factors [9, 34]. Therefore, considering the Yellow River Basin as the research area, the present study establishes a tourism eco-efficiency evaluation system from the perspective of geographical space, and reveals the temporal and spatial dynamic evolution characteristics of tourism eco-efficiency in the Yellow River Basin from 2009 to 2019. Additionally, the study uses the geographical and temporal weighted regression (GTWR) model to explore the influencing factors from the spatial and temporal perspectives, thereby providing a scientific decision-making basis for the implementation of high-quality development strategy in the Yellow River Basin and to realize sustainable development.

This study enriches the input-output theory and contributes to its development and application in the tourism industry. Researchers in the field of ecotourism can use the insights of this study to broaden their understanding of the economic interdependence within the tourism industry, helping them establish more accurate models and make better predictions. The study’s relevance to sustainable tourism development is crucial, particularly in developing countries where balancing economic growth with environmental and cultural preservation is a challenge. Policymakers, tourism boards, and NGOs in these countries can use the study’s findings as a reference to shape policies and practices that promote sustainable tourism, fostering economic growth while minimizing negative impacts on the environment and local communities.

## Material and methods

### Study area

The Yellow River Basin, which spans 9 provinces and regions (including Qinghai, Sichuan, Gansu, Ningxia, Inner Mongolia, Shaanxi, Shanxi, Henan, and Shandong), is situated in the northern part of China (95° 53’e –119° 0’e, 32° 10’n –41° 50’n). It covers an area of 795,000 km^2^, occupying approximately 8.3% of overall land area in China. In addition to China’s three gradient terrains, the basin also spans the following four geomorphic units, including the Qinghai–Tibet, Loess and Inner Mongolia Plateaus, as well as the North China Plain. The basin has both wet and dry climate. The middle and upper reaches are in semi-arid and sub-humid areas, and the ecological environment is fragile. As a vital ecological security barrier area in China, it is also the key area of the origin and progress of Chinese civilization. It is regarded as the birthplace of distinct regional cultures such as Hehuang, Guanzhong, Heluo, and Qilu, with diverse historical and cultural heritages, as well as rich and colorful natural landscapes such as deserts, grasslands, waterfalls and canyons, and rich tourism resources. As of October 2019, the Yellow River Basin has 20 world heritage sites, 4,140 A-level scenic spots, 112 national nature reserves, 250 national forest parks, 99 national geological parks, 51 national scenic spots, 1,451 national key cultural relics protection units, 678 traditional Chinese villages, and 136 historical and cultural cities, towns, and villages. The permanent population of the Yellow River Basin is approximately 400 million, with a GDP of 24,740.77 billion yuan, occupying 25.10% of the country’s total GDP. The total tourism revenue is 4,767.7 billion yuan, accounting for 11.82% of the total GDP of the basin.

**Fig 1:**
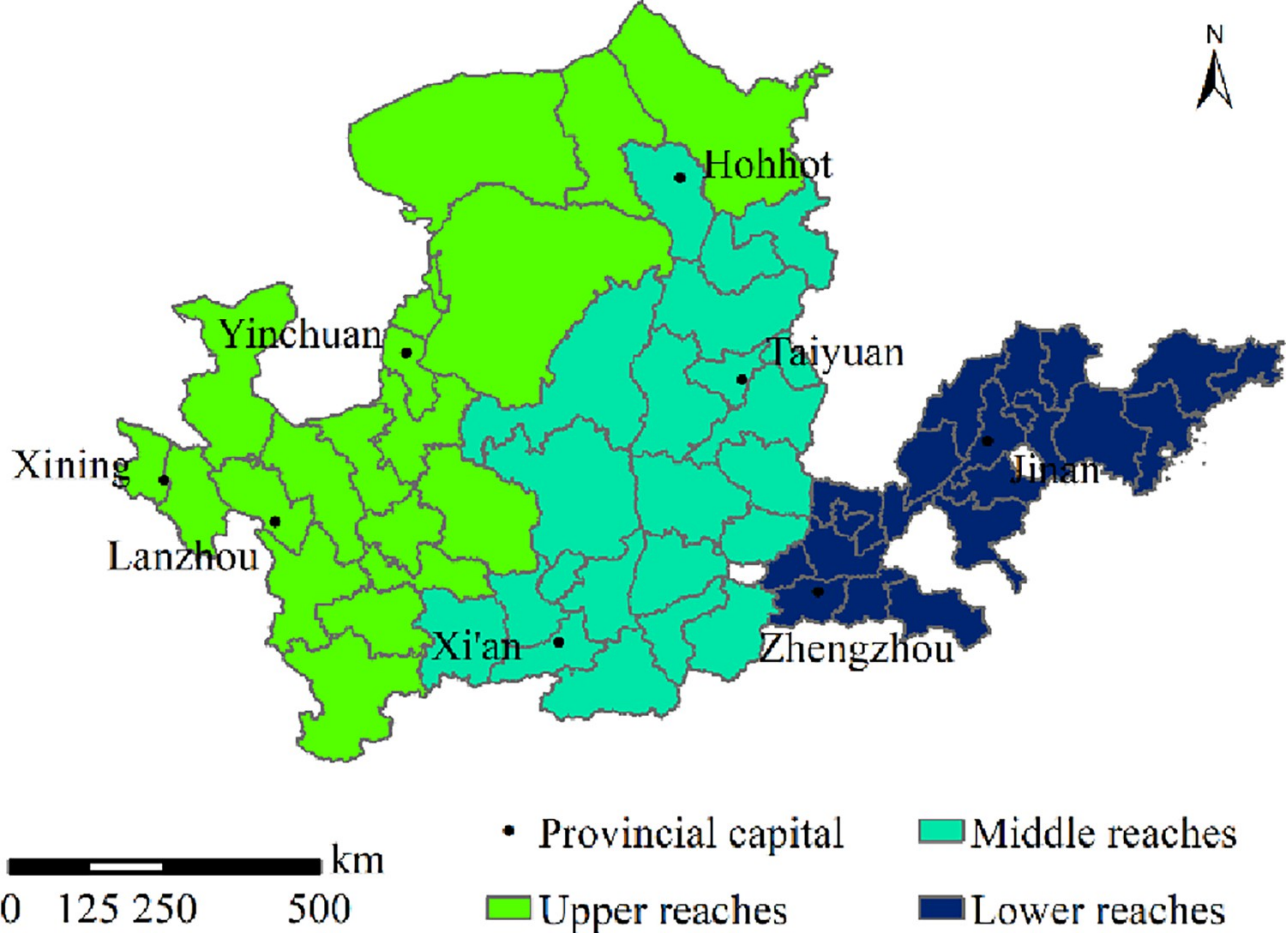
Study area.

China not only has a large population and a vast geographical area but is also rich in cultural tourism resources. It has 34 provincial-level administrative regions, with each region having a large geographical area and a little correlation with the Yellow River Basin. The study of tourism eco-efficiency in the Yellow River Basin at the provincial-level can produce biased results, and an accurate evaluation of the spatio-temporal variation of tourism eco-efficiency in the basin is impossible [38]. Therefore, this study considers a more accurate municipal scale to avoid the impact of tourism eco-efficiency of other provinces on the Yellow River Basin. Chinese scholars have conducted studies in this field mainly at the municipal scale [3, 26]. For data acquisition, 10 prefecture-level cities (prefectures or leagues) including Jiyuan City, Alxa League, Aba Tibetan and Qiang Autonomous Prefecture, Gannan Tibetan Autonomous Prefecture, and Linxia Hui Autonomous Prefecture have more missing data and have not been included in the study [39, 40]. Finally, 63 prefecture-level cities were identified (Fig 1). At the same time, the Yellow River Basin is a deep integration area of various geographical mutations [41], with great differences in nature and culture in different regions. To gain in-depth understanding of the tourism eco-efficiency of the Yellow River Basin, this study focuses on the Yellow River Basin by zoning and discusses the specific conditions of the upper, middle, and lower reaches.

### Index selection and data sources

Tourism eco-efficiency is a key index to measure the coordination between tourism economy and environment, and the premise of measuring it is to construct an appropriate input–output index. Existing studies have mainly focused on the basic factors such as capital, labor, and land, considering A-level scenic spots, the number of star-rated hotels, fixed asset investment, and tourism employees as input indicators; the total tourist reception and total tourism income as the expected output indicators; and tourism wastewater emission, tourism sulfur dioxide emission, tourism soot emission, and tourism CO_2_ emission as the non-expected output indicators. Based on the connotation of tourism eco-efficiency, input-output theory, and existing research results [26, 30, 34, 42, 43], and by considering the availability of data, in this study, tourism resources, tourism employees, total investment in fixed assets, tourism supply and demand service capacity (number of star-rated hotels and travel agencies), and environment and tourism energy consumption were selected as input indicators from the perspectives of resources, labor, capital, and tourism economic activity infrastructure. Tourism income and the number of tourists received were selected as expected output indicators, and tourism CO_2_ emission was selected as the non-expected output indicator. The details are as follows:

(1) Input index: ①Tourism resources constitute the core of tourism activities. Twelve indicators were selected from the perspectives of comprehensive tourism resources, humanistic tourism resources, and natural tourism resources to build a tourism resource evaluation index system, and the entropy weight method was used to quantify the selected indicators, determine the weights of various types of tourism resources, and calculate the tourism resource value of each city by weighted summing [44] (Table 1). ②Tourism practitioners are those who provide tourism services to tourists. As the number of tourism practitioners at municipal scale is difficult to obtain, urban tertiary industry practitioners were chosen as the representative sample. Although there is some error between this index and the input of actual elements of tourism, it basically includes the number of employment in related departments of tourism, which can fully demonstrate the comprehensiveness of urban tourism [45]. ③Fixed asset investment is a prerequisite for the normal development of tourism activities, which mainly includes the improvement of infrastructure, the introduction of advanced technology, and the development of new tourism products. The fixed asset price index is calculated with 2009 as the base period, the capital stock is calculated using the perpetual inventory method, and the depreciation rate is 9.6% [46]. ④Tourism supply and demand service capacity refers to the scale and number of tourists accepted in a region according to the standard. We find that the number of tourist attractions reflects the supply and demand capacity of local tourism. The number of star-rated hotels reflects the service capacity of urban tourism. The number of the selected star-rated hotels and tourist attractions stands for the service capacity of tourism supply and demand. ⑤Energy consumption. The normal operation activities of various tourism departments typically consume considerable energy. Because obtaining tourism energy consumption data of relevant departments at the municipal level is difficult, energy consumption is represented by the product of the ratio of the total tourism revenue of each city in regional GDP and municipal energy consumption.

**Table 1:** Tourism resource evaluation index system.

Target layer	Criterion layer	Index layer	Weight
**Tourism resources**	Comprehensive tourism resources (0.333)	5A tourist attraction	0.0891
		4A tourist attraction	0.0274
		3A and below tourist attractions	0.0276
	Humanistic tourism resources (0.333)	World heritage	0.2039
		National historical and cultural city	0.0774
		National historical and cultural town	0.1343
		National cultural relics protection units	0.0862
		National-level traditional village	0.1492
	Natural tourism resources (0.333)	National forest park	0.0826
		National geopark	0.1236
		National scenic spot	0.1606
		National nature reserve	0.1398

(2) Output index: ①Expected output. Direct output of tourism production is the one that can satisfy the demands and services of tourists during the traveling process. Tourism income is a direct measure of the benefits obtained from tourism business activities. In addition, the number of tourists received denotes the scale of tourism development. Therefore, tourism income (the sum of domestic and foreign tourism income, which is adjusted by the CPI index calculated in 2009 as the base period) and the number of tourists received (the sum of domestic and foreign tourists) are considered as output indicators [43]. ②Non-expected output. Although tourism is a low-carbon, low-consumption, and low-emission industry, the expansion of the tourism scale increases CO_2_ emission [47], which considerably affects the climate and environment [48]. Therefore, the present study considers CO_2_ emission of sectors related to tourism activities as the non-expected output.

Affected by the COVID-19 pandemic, the tourism data from 2020 to 2022 are not of reference significance. The input–output data of the current work are acquired from the China City Statistical Yearbook (2010–2020), Statistical Bulletin of National Economic and Social Development of Municipalities (2009–2019), Municipal Culture and Tourism Bureau, National Park website (http://gigy.com), and the website of China Cultural Administration (http://www.ncha.gov.cn).

### Research methods

#### Super-SBM model

Super-SBM model, which is proposed by Tone on the basis of SBM model, can be used to measure the combination of Super efficiency and SBM model [49]. This method has been extensively applied for measuring the tourism eco-efficiency [33, 37], and it can accurately calculate the value of tourism eco-efficiency by distinguishing the ranking problem of efficiency value of multiple research units as 1, reflecting the increase or decrease in the input or output of research units in the same proportion. The calculation formula is as follows:

$$\theta^{*}=\underset{\lambda ,s^{-},s^{+}}{\min}\frac{1+\frac{1}{m}\sum_{i=1}^{m}\frac{s_{i}^{-}}{x_{io}^{t}}}{1-\frac{1}{q+h}(\sum_{r=1}^{q}\frac{s_{r}^{+}}{y_{ro}^{t}}+\sum_{k=1}^{h}\frac{s_{k}^{-}}{b_{ko}^{t}})}$$ (1)

$$s.t.x_{io}^{t}\geq \sum_{t=1}^{T}\sum_{j=1,j\neq o}^{n}\lambda_{j}^{t}x_{ij}^{t}-s_{i}^{-}\;i=1,2,\ldots ,m;$$ (2)

$$y_{ro}^{t}\leq \sum_{t=1}^{T}\sum_{j=1,j\neq o}^{n}\lambda_{j}^{t}y_{rj}^{t}+s_{r}^{+}\;r=1,2,\ldots ,m;$$ (3)

$$b_{ko}^{t}\geq \sum_{t=1}^{T}\sum_{j=1,j\neq o}^{n}\lambda_{j}^{t}b_{kj}^{t}-s_{k}^{-}\;k=1,2,\ldots ,m;$$ (4)

$$\lambda_{j}^{t}\geq 0(\forall j),s_{i}^{-}\geq 0(\forall i),s_{r}^{+}\geq 0(\forall r),s_{k}^{-}\geq 0(\forall k)$$

In the equation, *x* represents input variables; *y* and *b* represent expected output and non-expected output variables, respectively; *λ* indicates the weight of selected elements; *o* represents decision unit; $s_{i}^{-}$, $s_{r}^{+}$, and $s_{k}^{-}$ represent relaxation variables of input, expected output, and non-expected output, respectively; θ* represents an efficiency value, with θ* > 1 suggesting an elevation in efficiency, θ* = 1 indicating no change in the efficiency, and θ* < 1 representing a decrease in efficiency. Based on previous studies [33, 50], the efficiency value was divided into five grades as follows: lower-efficiency area (<0.3), low-efficiency area (0.3–0.6), medium-efficiency area (0.6–0.9), high-efficiency area (0.9–1.2), and higher-efficiency area (>1.2).

#### Trend surface analysis

Trend surface analysis refers to a statistical method to fit the mathematical surface, which can show the spatial variation rule of the value [51]. This study used second-order polynomial for calculating the fitting value of tourism eco-efficiency and analyzing the spatial distribution pattern of tourism eco-efficiency value in the Yellow River Basin. The formula is as follows:

$$R_{i}(X_{i},Y_{i})=T_{i}(X_{i},Y_{i})+\varepsilon_{i}$$ (5)

In the equation, *R*_*i*_ represents the tourism eco-efficiency of a certain city, *R*_*i*_(*X*_*i*_, *Y*_*i*_) is the trend function, (*X*_*i*_, *Y*_*i*_) represents the geographical coordinates of a certain city, *T*_*i*_(*X*_*i*_, *Y*_*i*_) is the trend surface fitting value, and *ε*_*i*_ is the random error term.

#### Spatial autocorrelation analysis

Spatial correlation analysis is a powerful tool that can be used for detecting the forms and types of spatial effects. The degree of spatial agglomeration and dependence of geographic data can be assessed by establishing the spatial weight matrix to measure the similarity and difference of the same attribute values of different units in the space [36]. Spatial autocorrelation analysis is classified into global and local measures. In the present study, global *Moran*′*s I* index was used for testing the spatial agglomeration characteristics of tourism eco-efficiency in the Yellow River Basin, and *Local Moran′s I* index was adopted for testing local differences and agglomeration distribution characteristics. The calculation formulas are as follows:

$$Moran^{\prime }s\;I=\frac{n\sum_{i=1}^{n}\sum_{j=1}^{n}w_{ij}(x_{i}-\bar{x})(x_{j}-\bar{x})}{S^{2}\sum_{i=1}^{n}\sum_{j=1}^{n}w_{ij}}\;(i\neq j)$$ (6)

$$Local\;Moran^{\prime }s\;I=\frac{n(x_{i}-\bar{x})\sum_{j=1}^{n}w_{ij}(x_{j}-\bar{x})}{S^{2}}\;(i\neq j)$$ (7)

Where *n* represents the number of research units, *x* represents the tourism eco-efficiency of each city, $\bar{x}$ indicates the mean value of tourism eco-efficiency of each city, *S*^2^ is the square variance, and *w*_*ij*_ refers to the spatial weight matrix of geographical proximity. *Moran′s I* index ∈ [–1,1], implying that the closer the I is to 1, the stronger is the positive correlation degree between regions; the closer the I is to -1, the stronger the negative correlation degree between regions; and an I value close to 0 indicates that no spatial autocorrelation exists between regions.

#### Hot spot analysis

Hot spot analysis reflects the location of clustering in space of high or low value elements of data by calculating Getis-Ord $G_{i}^{*}y$ [52, 53]. In this study, this analysis was used to determine the location of spatial clustering of tourism eco-efficiency values in the Yellow River Basin, and the spatial autocorrelation results were verified and supplemented. The calculation formula is as follows:

$$G_{i}^{*}=\frac{\sum_{j=1}^{n}w_{ij}x_{j}-\sum_{j=1}^{n}x_{j}/n\sum_{j=1}^{n}w_{ij}}{\sqrt{\frac{\sum_{j-1}^{n}x_{j}^{2}}{n}-{\left(\bar{X}\right)}^{2}}\sqrt{\frac{\left[n\sum_{j-1}^{n}w_{ij}^{2}-{\left(\sum_{j-1}^{n}w_{ij}\right)}^{2}\right]}{n-1}}}$$ (8)

In the formula, $G_{i}^{*}$ statistically represents z score, *x*_*j*_ represents attribute value of factor j, *w*_*ij*_ represents the space weight between factor i and j, and n indicates the total number of factors. ${0<G}_{i}^{*}$ and $G_{i}^{*}$ higher than the critical value represent the hot spot area of tourism eco-efficiency; $G_{i}^{*}<0$ and $G_{i}^{*}$ lower than the critical value represent the cold spot area; and the rest is not significant area.

#### Elliptic standard deviation

The elliptic standard deviation was put forward by American sociologist Welty Lefever [54], which is employed to calculate the direction and distribution of data. In this study, elliptic standard deviation was used to quantitatively describe the spatial pattern evolution of tourism eco-efficiency values in the Yellow River Basin [4].

#### GTWR model

Based on the geographically weighted regression (GWR) model, GTWR model is an effective method to add time dimension and utilize spatiotemporal geographic information for improving the accuracy of regression results, thus accurately reflecting the spatial data relationship [55]. This study used GTWR model to explore the spatiotemporal influential factors of tourism eco-efficiency in the Yellow River Basin. The calculation formula is as follows:

$$y_{i}=\beta_{0}(u_{i},\;v_{i},\;t_{i})+\sum_{k=1}^{P}\beta_{k}(u_{i},\;v_{i},\;t_{i})x_{ik}+\varepsilon_{i}$$ (9)

Where, y_i_ suggests the value of tourism eco-efficiency of a certain city, (u_i_, v_i_, t_i_) represents the space-time coordinate of the ith city, β_k_(u_i_, v_i_, t_i_) stands for the KTH regression parameter of point *i*, x_ik_ represents the value of independent variable *x*_*k*_ at point *i*, and ε_i_ refers to the random error term.

## Results

### Temporal and spatial distributions of tourism eco-efficiency

**Fig 2:**
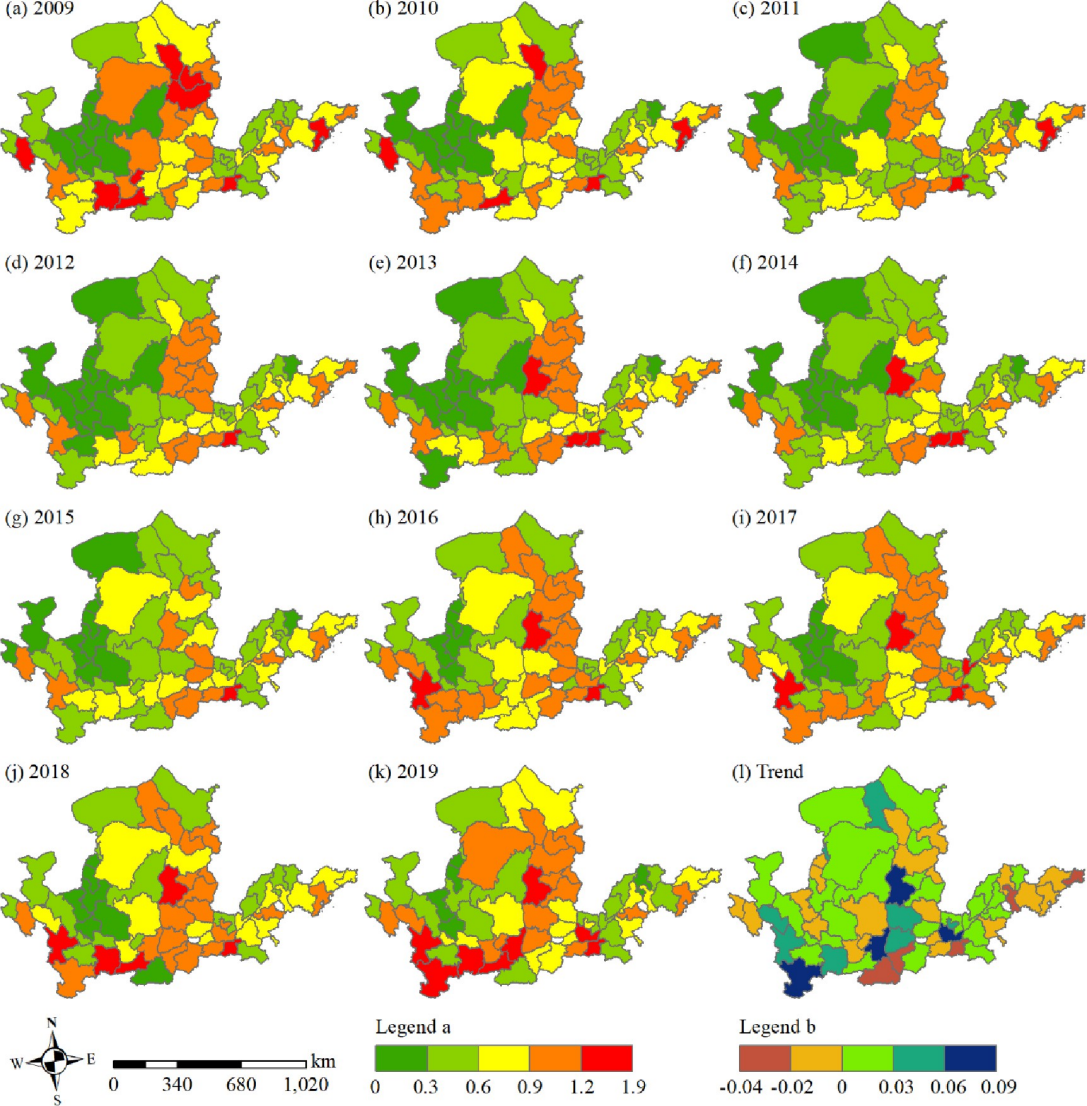
Spatial distribution pattern (a-k) and change trend (l) of tourism eco-efficiency in the Yellow River Basin from 2009 to 2019.

Temporal and spatial differences exist in tourism eco-efficiency of the Yellow River Basin (Fig 2). Overall, higher- and lower-efficiency areas were distributed in the midstream and upstream areas, respectively, whereas high-, medium-, and low-efficiency areas were dispersed in the downstream and upstream areas. The development trend of tourism eco-efficiency in the Yellow River Basin was excellent and exhibited an “oscillation period” from 2009 to 2014 and “improvement period” from 2015 to 2019. Among them, inefficient areas were distributed in Qinghai, Gansu, and Ningxia, and subsequently spread to Inner Mongolia and Shandong Province during 2011–2015 in a “decentralized” distribution pattern, and concentrated in Ningxia from 2016 to 2019. The number of efficiency zones increased gradually from 2009 to 2015 and decreased sharply from 2016 to 2019, indicating an overall decrease from 9 in 2009 to 5 in 2019, with the number of zones being maximum in 2012. Lower-efficiency areas are distributed around low-efficiency areas; these areas were concentrated in Gansu, Qinghai, Henan, and Shandong provinces from 2009 to 2015, expanded to Shaanxi, Inner Mongolia, and Shanxi from 2011 to 2015, narrowed from 2016 to 2019, and are presently distributed in Henan, Shandong, and Inner Mongolia, but more in Qinghai and Gansu. The number of efficiency zones in this area was large and increased from 15 in 2009 to 18 in 2019 and reached as high as 28 in 2014. The distribution of medium-efficiency areas was scattered within the whole basin but concentrated in the middle and lower reaches. During the period from 2009 to 2010, these efficiency areas were distributed in the upper, middle, and lower reaches, with most of them dispersed in the middle and lower reaches. The middle and lower reaches of the country also had a high distribution, and the number changed steadily in addition to the distribution in Tianshui and Lanzhou in 2015 and 2017 from 2011 to 2019. In addition, the number of higher-efficiency areas increased from 15 in 2009 to 17 in 2019, and they were concentrated in the midstream. From 2014 to 2015, the number was small, and the distribution was dispersed, whereas in the rest of the years, they were concentrated in Shanxi. High-efficiency areas exhibited a scattered distribution, and their number was small. Kaifeng has been a part of high-efficiency areas. According to the change trend shown in Fig 2I, the high value areas of efficiency change trend in cities of the Yellow River Basin are distributed in Longnan City, Weinan City, Xinxiang City, and Lvliang City, with positive and negative areas exhibiting a “decentralized” distribution pattern, and the distribution range of positive areas being greater than that of negative areas.

#### Time variation of tourism eco-efficiency

**Fig 3:**
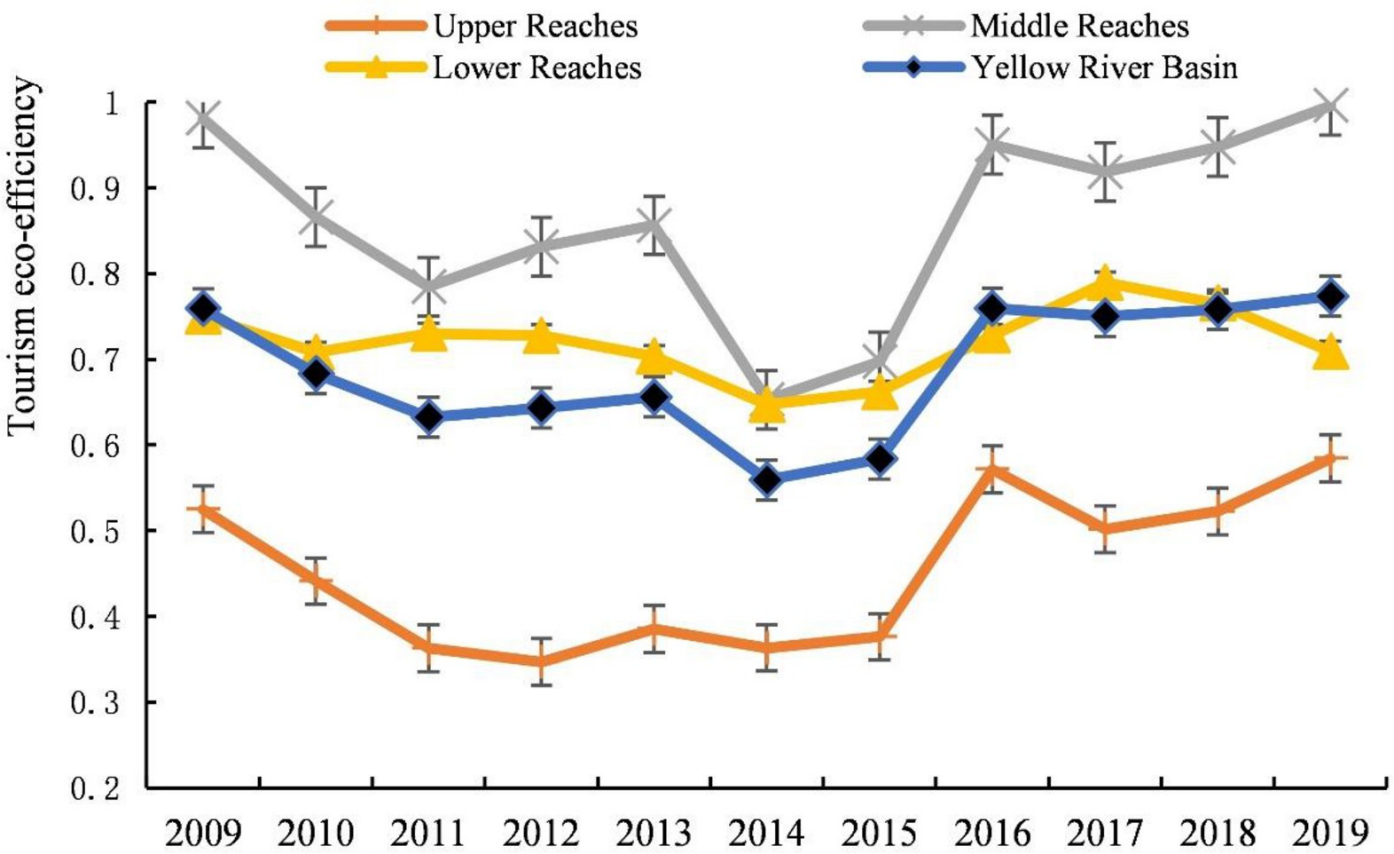
Temporal changes of tourism eco-efficiency in the Yellow River Basin from 2009 to 2019.

Over years, the tourism eco-efficiency of the Yellow River Basin has varied considerably, going through the “oscillation period” from 2009 to 2014 and the “improvement period” from 2015 to 2019 (Fig 3). The “oscillation period” exhibited a fluctuating downward trend and a large amplitude, and the efficiency value in this stage was distributed between 0.56 and 0.77. The “improvement period” included a rapid increase from 2015 to 2016 and a stable development from 2017 to 2018. From low to high, the efficiency values of the upper, middle, and lower reaches of the basin were in the order: middle reaches > lower reaches > upper reaches. Additionally, the tourism efficiency value of upstream areas presented a downward trend from 0.53 in 2009 to 0.36 in 2014, with the decline being large from 2009 to 2011 and stable from 2011 to 2015. From 2015 to 2019, a fluctuating upward trend, elevating from 0.38 in 2015 to 0.58 in 2016 was observed, indicating a large increase, decreasing first and subsequently rising from 2016 to 2019. The fluctuation trend of the efficiency value in the middle and lower reaches was close to the overall trend of the Yellow River Basin, while the range within the middle reaches was obvious, with the lower reaches being more stable.

**Fig 4:**
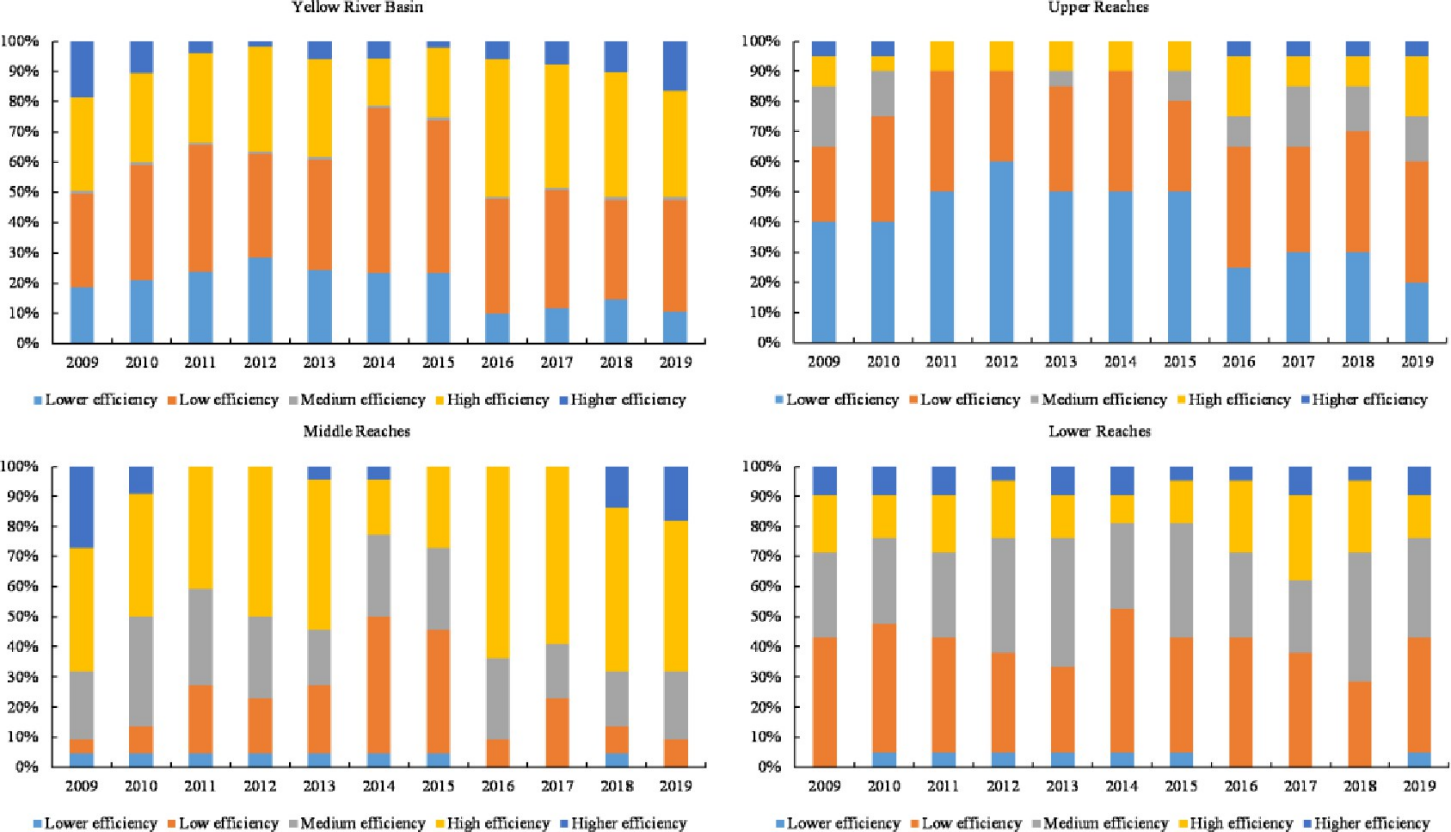
Time changes of the proportion of eco-efficiency of various tourism levels in the Yellow River Basin from 2009 to 2019.

Considerable regional differences were observed in the proportion of tourism eco-efficiency among various levels. The proportion of high- and higher-efficiency areas in the Yellow River Basin exhibited an increasing trend, whereas the proportion of low- and lower-efficiency areas exhibited a decreasing trend (Fig 4). Low- and high-efficiency area occupied a significant proportion, followed by lower-efficiency, higher-efficiency, and medium-efficiency area. The highest proportion of low-efficiency area was 44% in 2014, whereas lower-efficiency areas exhibited a decreasing trend from 14% in 2009 to 8% in 2019. The efficiency levels of upper, middle, and lower reaches were lower, high, and low, followed by low, high, and middle, respectively. The proportion of other grades was small. The proportion of high-efficiency areas in the upper reaches gradually increased from 3% in 2009 to 6% in 2019, whereas the proportion of lower-efficiency areas gradually decreased from 13% in 2009 to 6% in 2019. The proportion of high- and higher-efficiency areas in the middle and lower reaches increased, whereas that of the lower-efficiency areas progressively decreased.

#### Spatial change of tourism eco-efficiency

**Fig 5:**
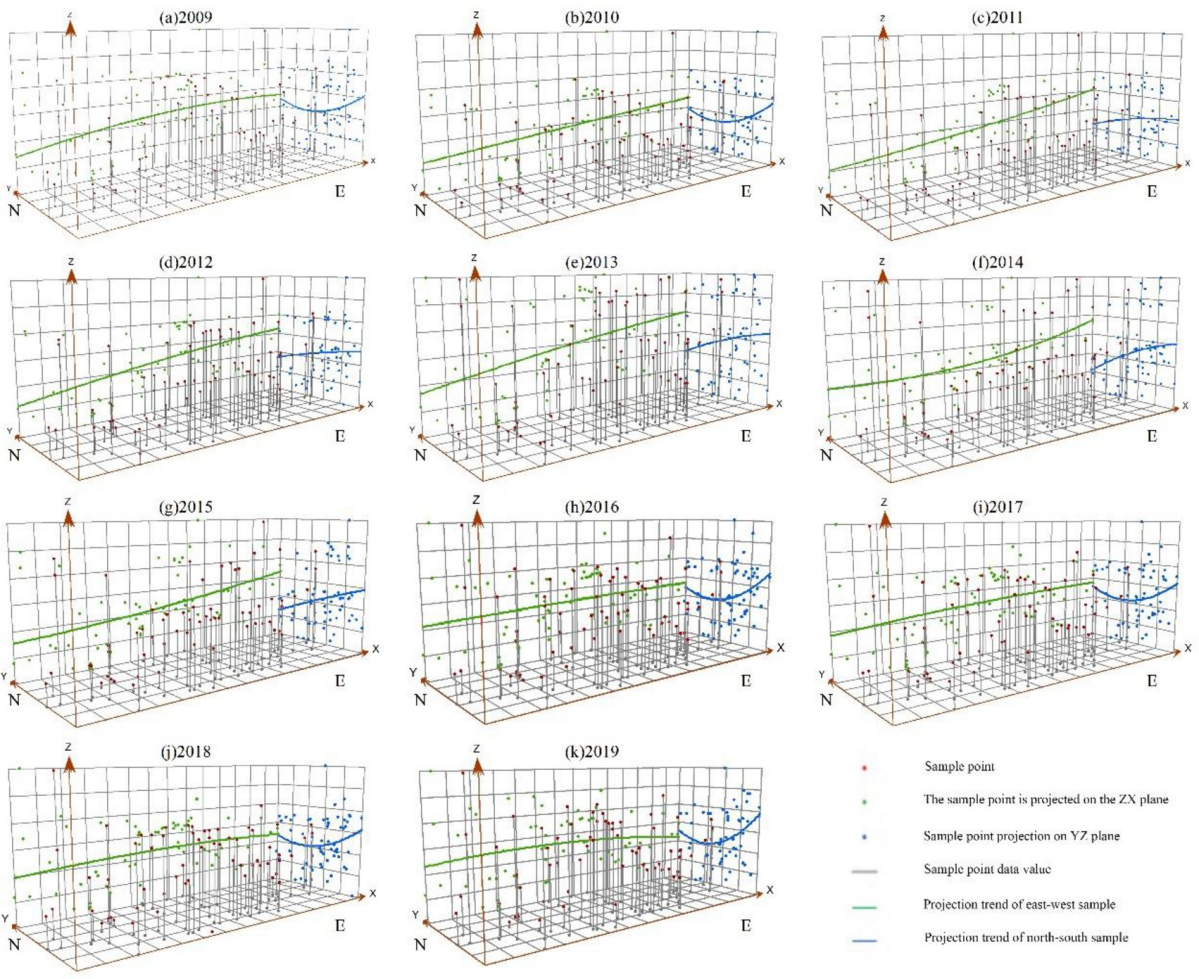
Trend surface analysis of tourism eco-efficiency in the Yellow River Basin from 2009 to 2019.

The spatial differentiation of tourism eco-efficiency in the Yellow River Basin was obvious, with a spatial structure of “high in the east and low in the west” in the east–west direction. Additionally, the slope first increased and subsequently reduced. The north–south direction presented a “U” shaped parabolic feature extending from north to south (Fig 5). The existing difference in efficiency values between the east and west directions first widened and subsequently narrowed. From 2009 to 2014, a straight line tilted from west to east and then gradually inclined. From 2016 to 2018, the straight line gradually became flat, and in 2019, a flat curve was observed, with low values at the east and west ends and slightly higher values in the middle. Additionally, the difference in efficiency values between the north and south directions exhibited a trend of widening, narrowing, and expanding. No change was observed in the spatial trend surface from 2009 to 2010, but a significant change was observed from 2011 to 2015, with high degrees of variation at the north and south ends, evolving from a U-shaped parabola to a straight line. From 2016 to 2019, the south and north ends rapidly increased, evolving from a straight line to a U-shaped parabola, with a steeper curvature compared with that from 2009 to 2010.

### Evolution characteristics of the spatial pattern of tourism eco-efficiency

#### Spatial autocorrelation analysis

The tourism eco-efficiency of the Yellow River Basin shows dramatical spatial interdependence(Table 2). During the study period, Moran’s I was positive, and Z-scores were greater than 1.96, with P values being less than 0.01, thereby rejecting the null hypothesis at the significance level of 1%. In accordance with this result, the spatial distribution of tourism eco-efficiency in the Yellow River Basin from 2009 to 2019 exhibited significant spatial interdependence, with considerable club convergence characteristics. Cities having similar tourism eco-efficiency values exhibited spatial agglomeration; cities with high-efficiency values were adjacent to cities with high-efficiency values. Besides, cities with low-efficiency values were adjacent to those with low-efficiency values.

**Table 2:** Global Moran’s I of tourism eco-efficiency in the Yellow River Basin from 2009 to 2019.

Time	Moran’s I	Z-Score	P-Value
**2009**	0.214	3.272	0.001
**2010**	0.202	3.126	0.002
**2011**	0.233	3.556	0.000
**2012**	0.280	4.212	0.000
**2013**	0.284	4.251	0.000
**2014**	0.169	2.640	0.008
**2015**	0.164	2.688	0.007
**2016**	0.253	3.837	0.000
**2017**	0.251	3.802	0.000
**2018**	0.214	3.290	0.001
**2019**	0.229	3.486	0.000

**Fig 6:**
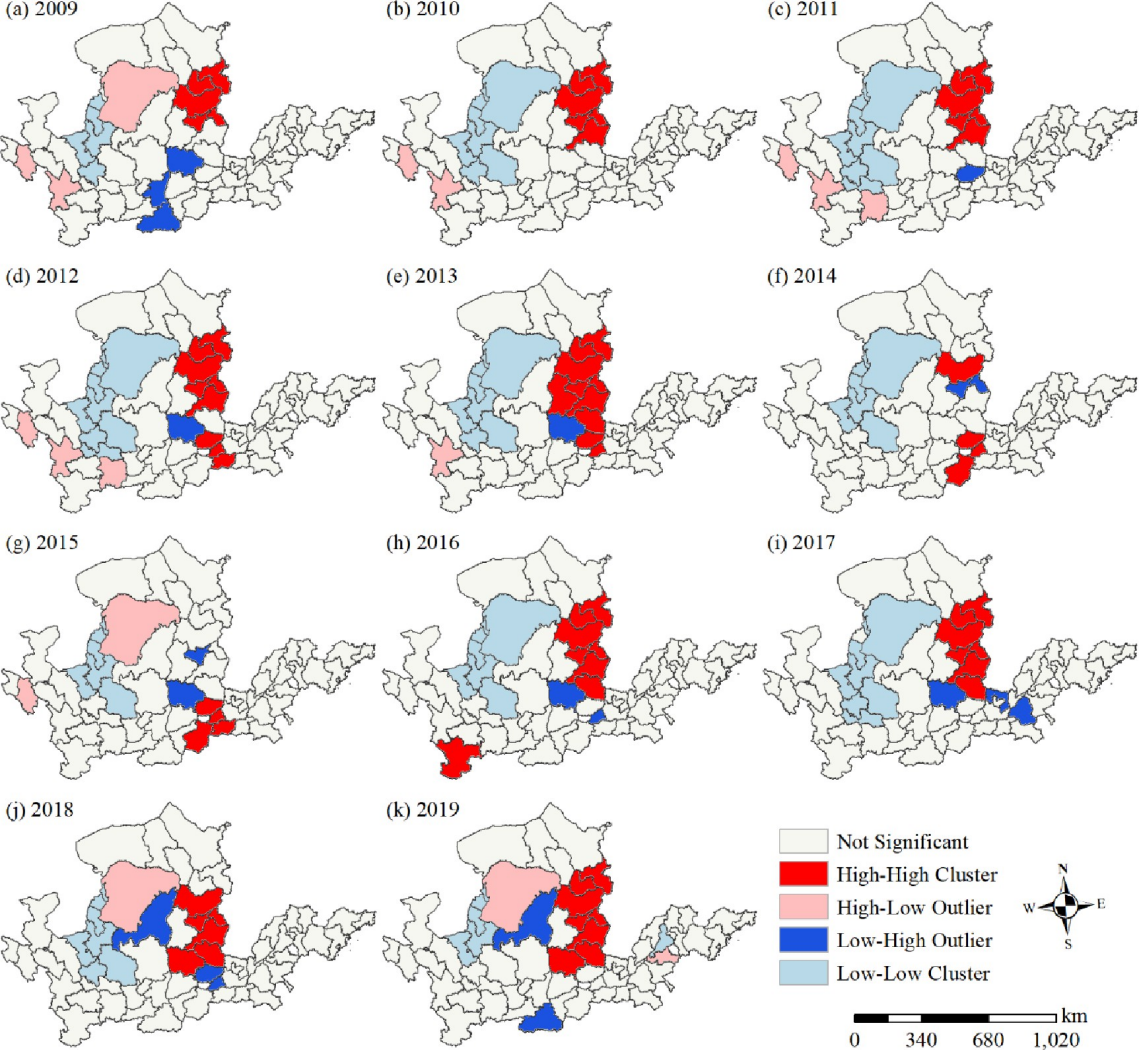
Local spatial correlation pattern of tourism eco-efficiency in the Yellow River Basin.

Local spatial correlation characteristics were analyzed to determine spatial differences in the tourism eco-efficiency in the Yellow River Basin (Fig 6). Consistent with the investigation of local spatial correlation characteristics, the formation of tourism eco-efficiency in the Yellow River Basin is characterized by H–H and L–L types, supplemented by H–L and L–H types, with concentrated and contiguous distribution of H–H and L–L types, and scattered distribution of H–L and L–H types. From 2009 to 2013 and from 2016 to 2019, the H–H area of tourism eco-efficiency in the Yellow River Basin was concentrated and distributed in Shanxi Province in the middle reaches of the Yellow River Basin. Additionally, the spatial distribution range continued to expand, and the agglomeration effect became increasingly obvious. The distribution of H–L and L–H areas was found to be scattered, mostly within the upper and middle reaches of the Yellow River Basin. The number of cities in this state was found to be small. Moreover, the spatial distribution of the L–L region was stable, concentrated, and contiguous in the upper reaches of the Yellow River Basin, which include Ningxia, Inner Mongolia, Gansu, and Qinghai provinces.

#### Hot spot analysis

**Fig 7:**
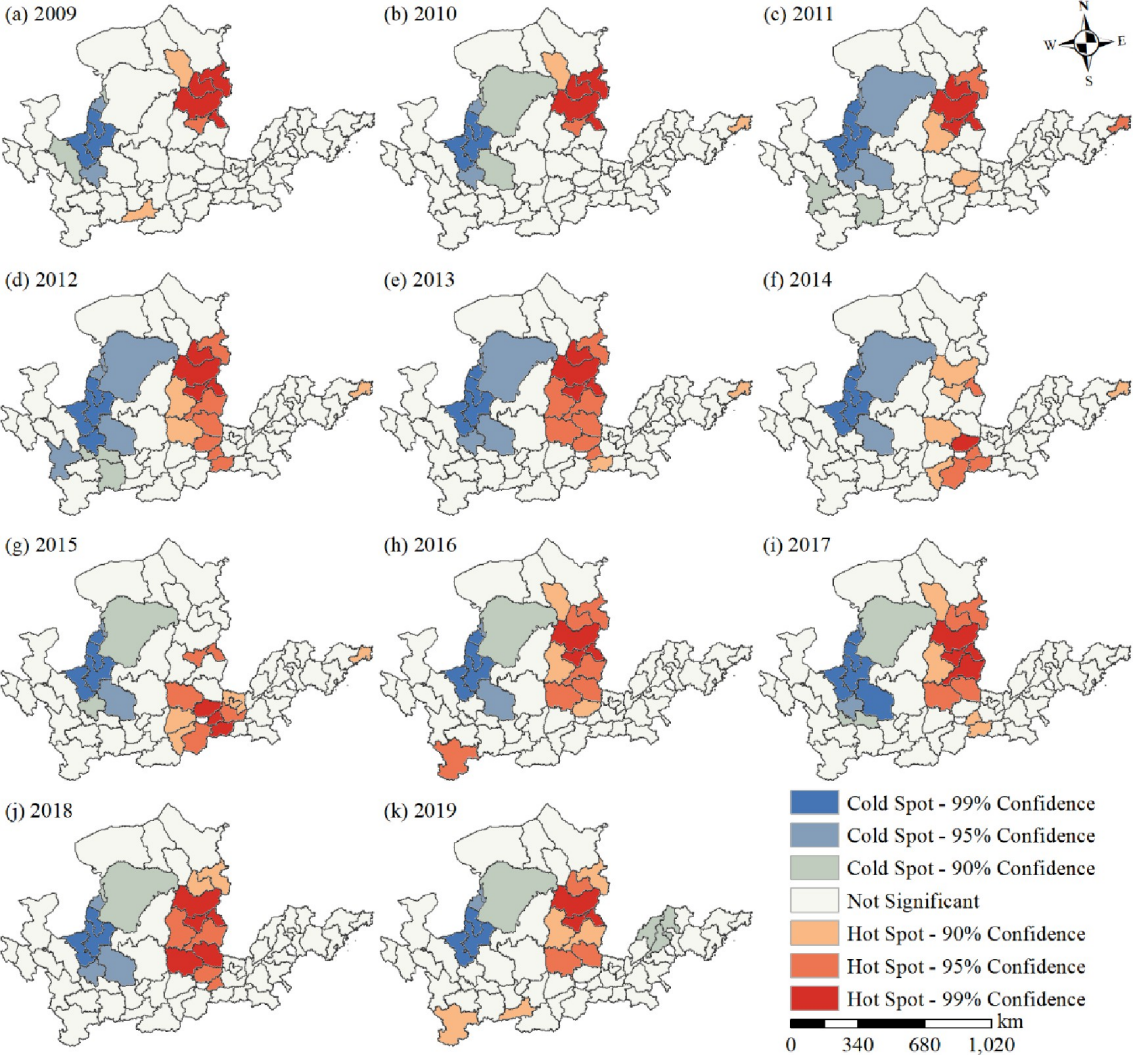
Spatial distribution of hot spots of tourism eco-efficiency in the Yellow River Basin.

The spatial distribution of cold and hot spots in the tourism eco-efficiency of the Yellow River Basin was significantly regional(Fig 7). The hot and cold spots in the Yellow River Basin were distributed within the middle and upper reaches of the Yellow River Basin, respectively. Significant hot and cold spots were distributed in contiguous areas, whereas a few areas exhibited a scattered distribution. The sub-significant hot and cold spots were distributed around the periphery of the significant hot spots and cold spots. From 2009 to 2013 and from 2016 to 2019, significant hot spots were distributed in Datong, Shuozhou, Xinzhou, and Yangquan, whereas sub-significant hot spots were distributed around the significant hot spots, gradually expanding their area. From 2014 to 2015, significant hot spots shifted to Jincheng and Shanxi, and their distribution scope narrowed. From 2009 to 2019, significant cold spots were distributed in Shi zuishan City, Yinchuan City, Wuzhong City, Zhongwei City, and Guyuan City. In 2019, the secondary cold point area shifted downstream to Binzhou City, Dezhou City, and Jinan City.

#### Elliptic standard deviation analysis

**Fig 8:**
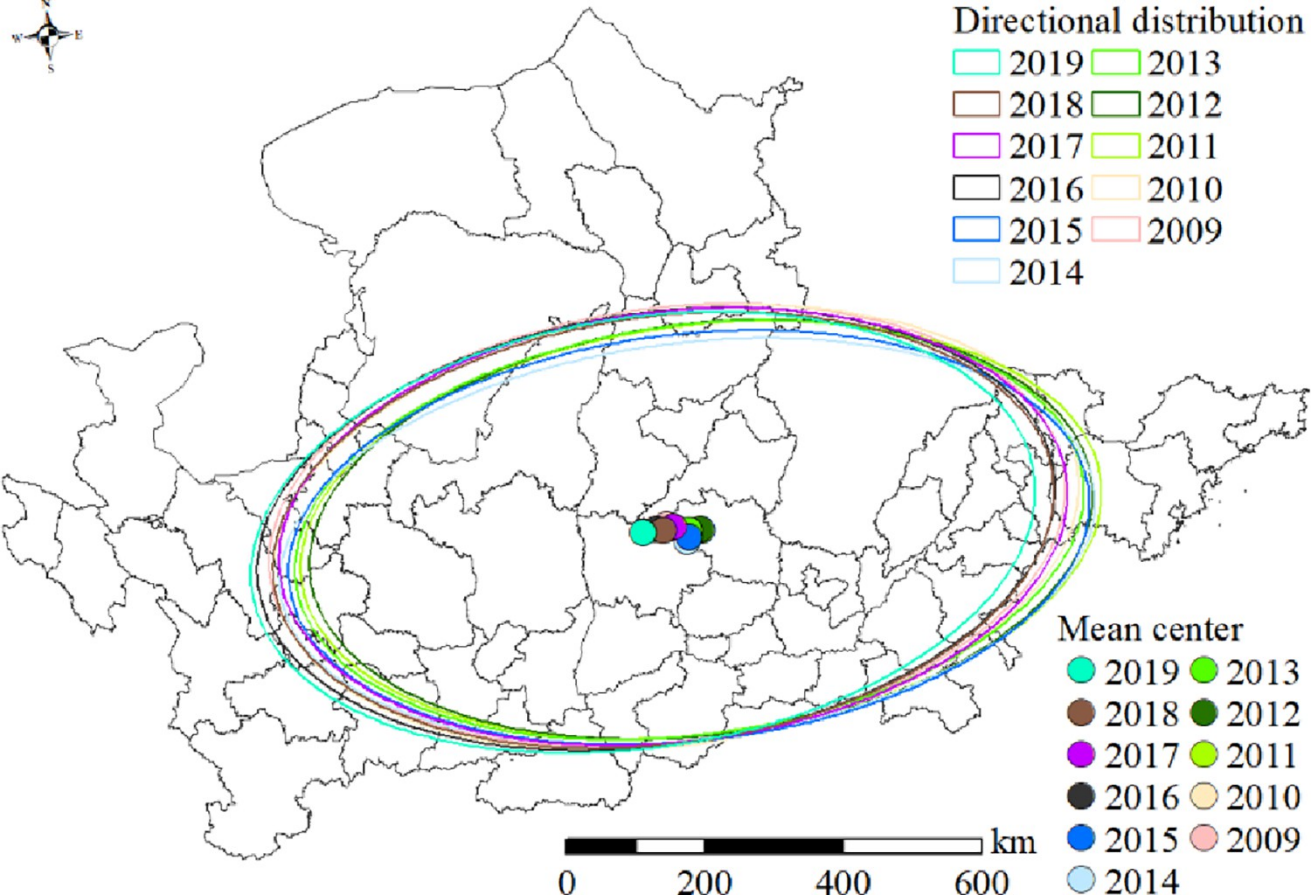
Spatial distribution of standard deviation ellipse and mean center of tourism eco-efficiency in the Yellow River Basin.

The standard deviation ellipse of tourism eco-efficiency in the Yellow River Basin exhibited a narrow distribution, showing an east to north to west to south trend (Fig 8). From 2009 to 2019, the standard deviation ellipse first expanded to the northeast and subsequently shifted to the southwest. From 2009 to 2017, the easternmost part of the standard deviation ellipse was within Dongying City, and only in 2018 and 2019, the easternmost part of the standard deviation ellipse appeared in Zibo City. The trajectory change of the average center point was not significant and concentrated in the northwest direction of Changzhi City from 2009 to 2015. From 2015 to 2019, the center point exhibited significant changes, gradually shifting from Changzhi City to the junction of Linfen City and Jinzhong City.

**Fig 9:**
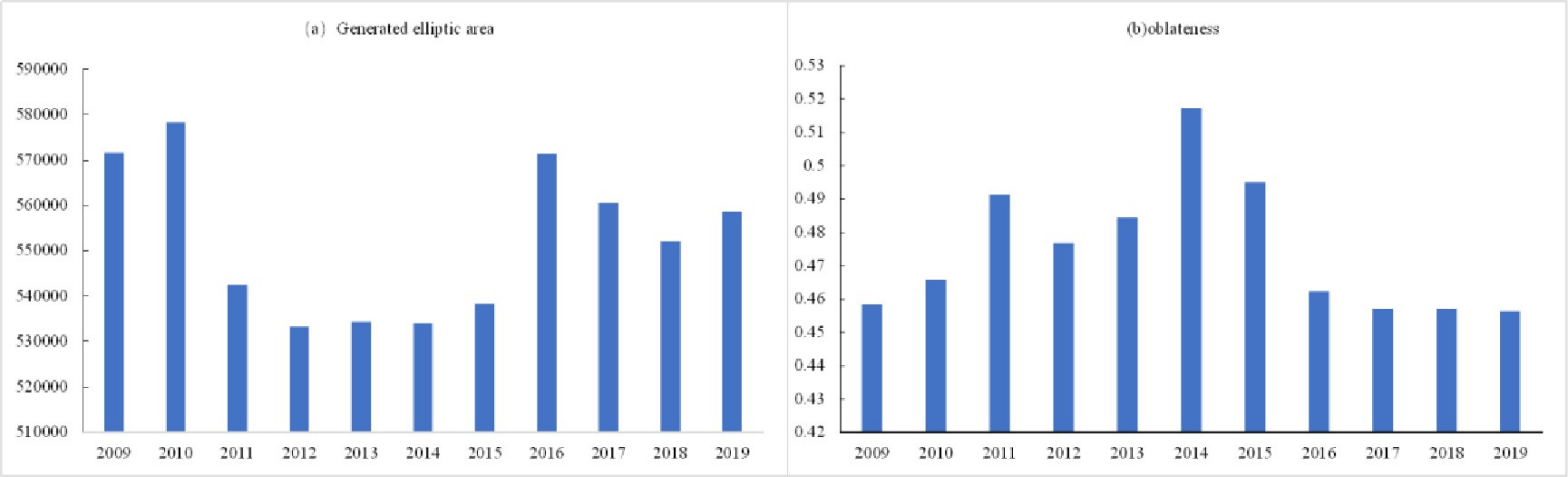
Standard deviation ellipse of tourism eco-efficiency in the Yellow River Basin generates elliptic area and flatness change.

The standard deviation of tourism efficiency in the Yellow River Basin was found to change significantly in the elliptical area, exhibiting a trend of first decreasing and subsequently increasing (Fig 9A). The elliptical area decreased sharply from 2010 to 2011 and increased from 2015 to 2016. No significant changes were observed during 2009–2010, 2011–2015, and 2016–2019. The elliptical areas during 2009–2010 and 2016–2019 were considerably larger than those during 2011–2015. The flattening value first exhibited a fluctuating trend and subsequently a decreasing trend(Fig 9B). The flattening rate increased gradually from 2009 to 2011 and 2012 to 2014 and subsequently began to decline from 2015 to 2019. The flattening rate was high in 2011, 2014, and 2015, which indicated a significant change in the efficiency values compared with other years.

### Analysis of factors affecting tourism eco-efficiency

#### Model variable selection

The investigation of the temporal and spatial patterns of tourism eco-efficiency in the Yellow River Basin indicated that due to the interaction among different factors, obvious temporal and spatial heterogeneity exists in the tourism eco-efficiency of 63 cities. Based on the principles of selecting influential factors and data availability of existing research results, the present study considers the panel data of cities from 2009 to 2019. The GTWR model was adopted for analyzing the factors affecting the tourism eco-efficiency of cities at various time points, considering the tourism eco-efficiency value as a dependent variable and social and natural factors of cities as explanatory variables, which include the economic development level (per capita GDP), investment in fixed assets (the price index of fixed assets calculated based on 2009 is calculated by the sustainable inventory method, and the depreciation rate is 9.6%), the intensity of tourism industry agglomeration (the level of tourism industry agglomeration is calculated by the location entropy index, and the domestic and foreign income of tourism in each city accounts for the proportion of GDP in each city/the domestic and foreign income of tourism in the whole country accounts for the proportion of GDP in the whole country), science and technology investment (total regional science and technology and education expenditure/regional GDP), education level (number of students in Colleges and universities), market opening level (total import and export volume of each city/regional GDP), tourism attractions (Table 1), transportation level (road network density of each city), environmental regulation (government investment in environmental pollution control/regional GDP), and regional supply and demand service capacity (the sum of the number of star hotels and class a scenic spots). The aforementioned explanatory variable index data were acquired from the China Urban Statistics Yearbook 2010–2020, the Statistical Bulletin on National Economic and Social Development of cities 2009–2019, and the Bureau of Culture, Radio, Television and Tourism.

#### Data testing and model results

Before using the GTWR model, the selected explanatory variables were standardized. The multicollinearity test of the standardized data was performed through multiple linear regression analysis in SPSS 4.0 software, and the selected variables with VIF values greater than 10 were excluded. Finally, six indicators, namely market opening intensity (X1), science and technology level (X2), environmental regulation (X3), tourism industry agglomeration intensity (X4), economic development level (X5), and transportation level (X6), were selected as the final explanatory variables of the GTWR model.Table 3 presents the parameters associated with the results of the spatio-temporal geographically weighted regression. In terms of the goodness of fit, it is corrected to be close to 0.71, indicating that the GTWR model can be used to measure the impact of the explanatory variables on the dependent variable.

**Table 3:** Related parameters of GTWR.

Number	Parameter	Price
**1**	Bandwidth	70
**2**	Residual Squares	199.595
**3**	Sigma	0.537
**4**	AICc	1348.36
**5**	R^2^	0.712
**6**	Adjusted	0.710
**7**	Spatiotemporal Distance Ratio	0.279

**Fig 10:**
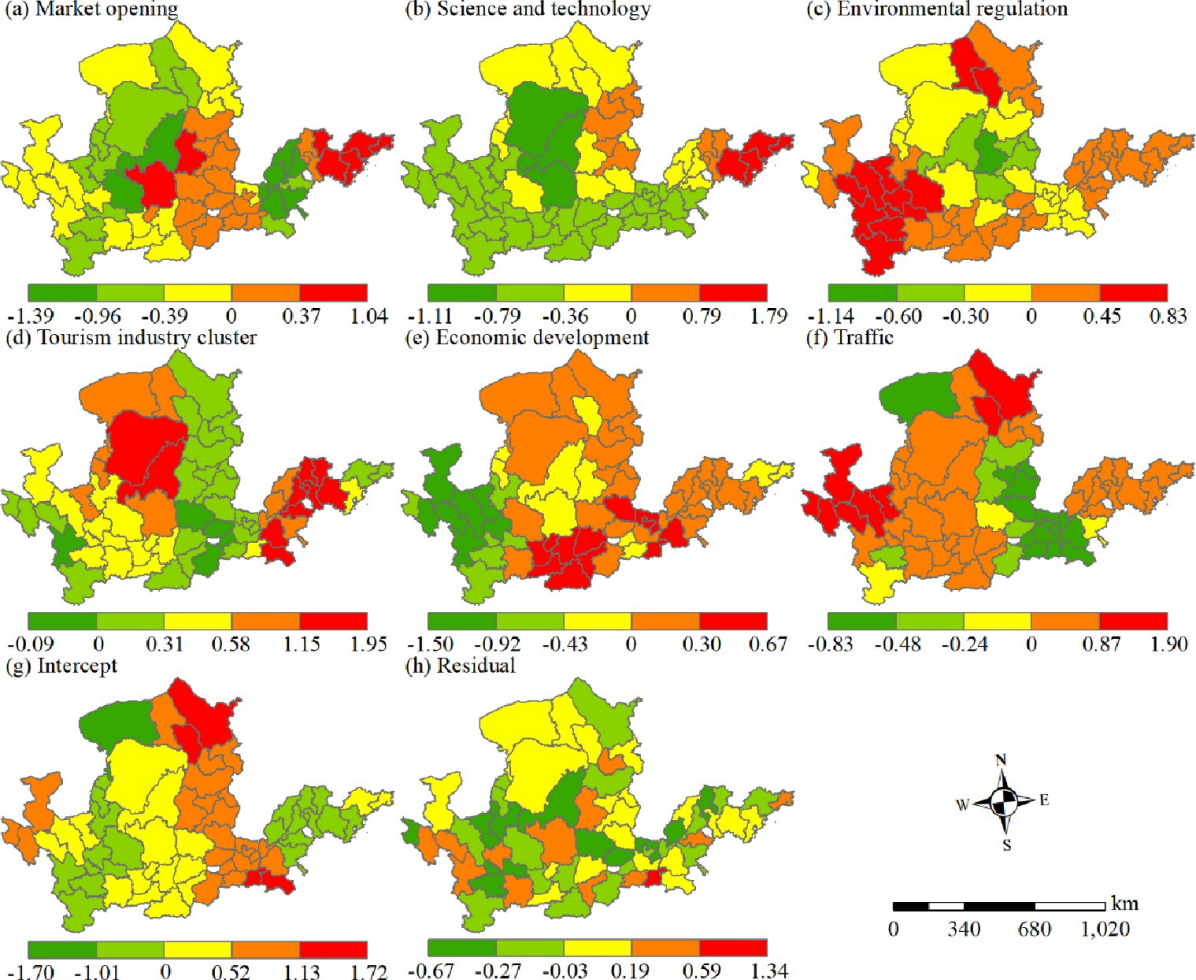
Spatial distribution of the regression coefficients of influencing factors of tourism eco-efficiency in the Yellow River Basin from 2009 to 2019.

Analysis of influencing factors. *(1) Spatial distribution of GTWR tourism eco-efficiency explanatory factor coefficients*. The explanatory factors for tourism eco-efficiency exhibited significant spatial heterogeneity (Fig 10), and obvious individual differences were observed in the impact of each factor on the efficiency value. According to the absolute value of the impact degree, the factors can be ordered as follows: economic development level > market openness > tourism industry agglomeration intensity > environmental regulation > science and technology level > transportation level. The impact of market opening on efficiency was negative, with the positive effect being low. In the Yellow River Basin, the greater the intensity of market opening, the lower the eco-efficiency of tourism, and the absolute value of its coefficient was the largest in the midstream and upstream but smallest in the downstream. Market opening negatively affected the midstream and upstream regions, and the effect was weaker in the downstream regions. The effect of science and technology on tourism eco-efficiency was positive in some cities in Shandong and Shanxi but negative in the remaining areas. The absolute value of its regression coefficient showed a pattern of diminishing around the high value areas of Shandong Peninsula and “three energy males” (Ordos, Yulin, Yan’an), and the impact of science and technology decreased from Shandong Peninsula and “three energy males” to the surrounding. The effect of environmental regulation on the efficiency value varied considerably in space, and the spatial pattern of positive-negative-positive effect alternating from southwest to northeast had a coefficient range of −1.14 to 0.84; the absolute coefficient value was high in Gansu, Ningxia, Inner Mongolia, and Lvliang City in the upper reaches of the Yellow River Basin, implying that environmental regulation has the greatest impact on these areas, whereas its impact in the rest of the areas is weak. The effect of tourism industry agglomeration intensity on the efficiency value differed considerably in space, and its coefficient ranged from −0.09 to 1.95. In the downstream areas, the tourism industry agglomeration coefficient was positive, indicating a positive effect on the efficiency value, and the absolute value of the coefficient was large, which indicated that tourism industry agglomeration considerably affected the efficiency value. Within the middle reaches, the agglomeration coefficient value of tourism industry was small, and the influence on the efficiency value was positive, which indicated that the positive impact on the middle reaches was weak. The spatial difference in the upstream region was large, and the coefficient value gradually increased from south to north, which indicated that the impact of tourism industry agglomeration on the upstream gradually elevated from south to north. The economic development level was found to have the greatest effect on tourism eco-efficiency; its regression coefficient was positive and the value was the largest. The spatial distribution exhibited negative distribution in the upper reaches and positive distribution in the middle and lower reaches, and the distribution range of positive areas was greater than that of negative areas. The effect of traffic level on tourism eco-efficiency was the smallest, with its coefficient value ranging from −0.83 to 1.90; the coefficient value of the upstream and downstream areas was large and positive, indicating that the positive effect of traffic on upstream and downstream areas was strong. The coefficient value of the middle reaches was negative, indicating a negative impact of traffic on the middle reaches.

**Fig 11:**
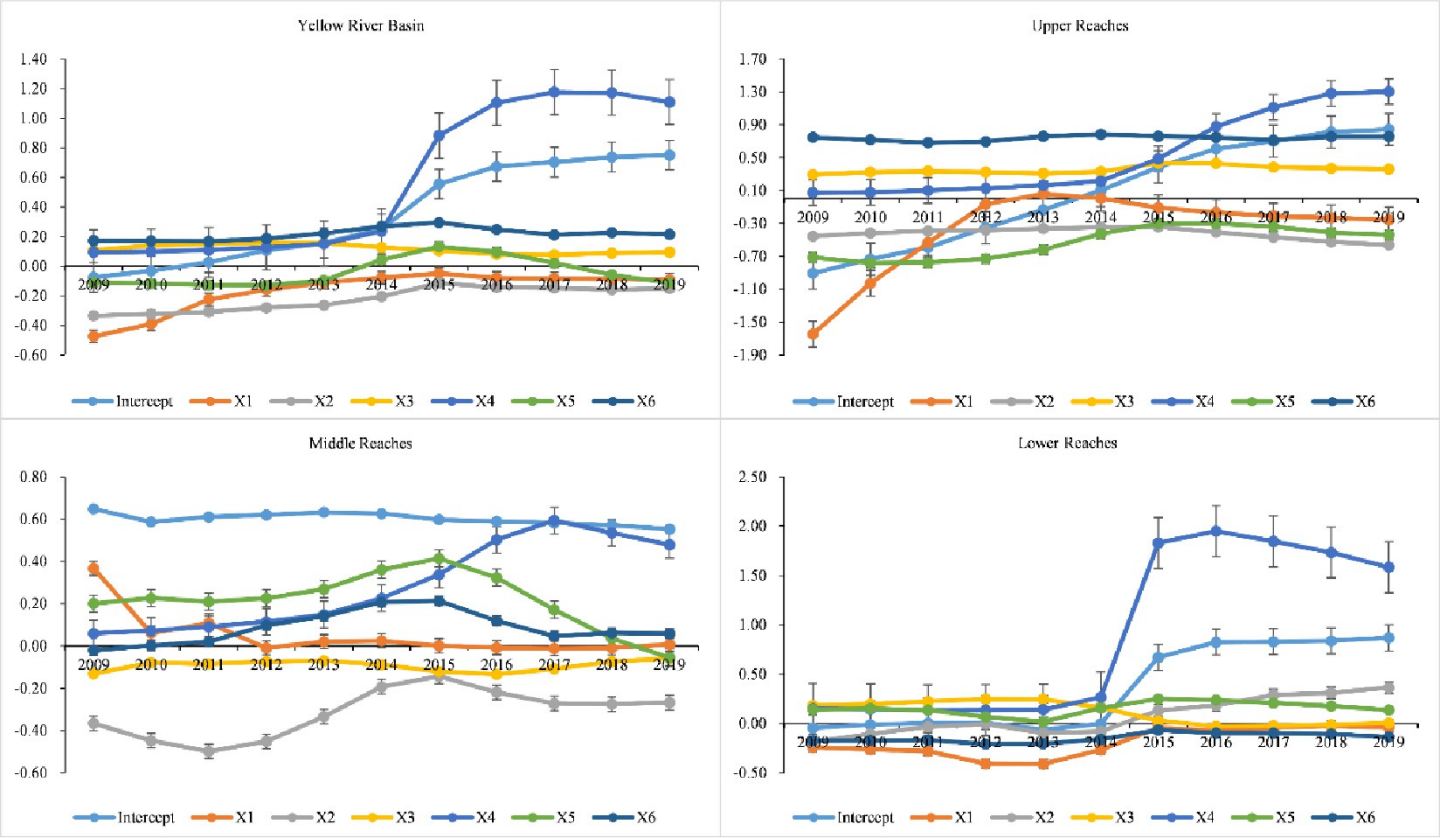
Temporal variation of the regression coefficient of tourism eco-efficiency in the Yellow River Basin from 2009 to 2019.

*(2) Time evolution of explanatory factors*. The influence of explanatory factors on the tourism eco-efficiency of the Yellow River Basin varied across different time periods (Fig 11). The change trend of the coefficients of the explanatory factors, namely transportation level, science and technology level, economic development level, market opening intensity, and environmental regulation, was stable, and the intensity factor and intercept of tourism industry agglomeration exhibited a “stable period” from 2009 to 2014 and a “rapid upward period” from 2016 to 2019. The coefficients of market opening intensity and science and technology were negative. The coefficient of economic development level was negative in 2009–2014 and 2017–2019 but positive in 2014–2017. The intercept was negative from 2009 to 2011 and positive from 2012 to 2019. The coefficients of environmental regulation, transportation level, and tourism agglomeration intensity were positive. The coefficients of upstream science and technology level and economic development level were negative and did not change considerably. The coefficient of market openness was negative, which increased rapidly from 2009 to 2013 and decreased gradually from 2004 to 2019. The intercept coefficient increased rapidly, and it was negative from 2009 to 2014 and positive from 2005 to 2019. The traffic level and environmental regulation coefficients were positive and did not change significantly during the study period. The coefficient of tourism agglomeration intensity was positive; it did not change significantly from 2009 to 2014 but increased rapidly from 2015 to 2019. The market opening coefficient in the middle reaches was positive, which showed a fluctuating decreasing trend from 2009 to 2012, with a large amplitude, but remained stable from 2013 to 2019. The science and technology coefficient was negative, showing a horizontal “S” shape from 2009 to 2019, which indicates a decrease, followed by an increase and then again a decrease. The environmental regulation coefficient was negative and did not change considerably. The coefficients of tourism agglomeration intensity, transportation level, and economic development level were positive, exhibited a trend of increasing first and decreasing subsequently. The intercept value was positive and large, and no obvious change trend was observed. From 2009 to 2014, the change of downstream tourism agglomeration intensity and intercept coefficient was stable and the absolute value was small, which increased rapidly in 2015 and subsequently became stable, and its absolute value was large. The other coefficients did not change considerably, and their absolute value was small.

## Discussion

### Spatial and temporal distribution characteristics of tourism eco-efficiency

The Yellow River Basin’s tourism eco-efficiency was low, with uneven spatial distribution. The overall development revealed a fluctuating upward trend, and differences were observed in the development trends among various regions. The tourism industry in this basin was dominated by the extensive development model of resource consumption, without forming an intensive and low-carbon tourism development model driven by scientific and technological innovation, as well as the positive interaction mechanism between tourism economy and ecological protection [56]. From 2009 to 2014, tourism became a strategic pillar industry of the national economy. Governments and enterprises throughout the Yellow River Basin began developing the tourism industry vigorously. However, most tourism projects are blind expansion, with disorderly development and construction, which exhibited the characteristics of “high input, light quality and low output” [44]. Moreover, the environmental problems related to tourism have become increasingly prominent, and tourism eco-efficiency is not high (Figs 3 and 4). The spatial distribution of tourism eco-efficiency within the basin was uneven, displaying a spatial structure of “high in the east and low in the west” (Fig 5) with obvious regional characteristics [3]. The economic development level is a critical factor driving the development of tourism eco-efficiency [2]. Economically developed regions possess complete resource endowment elements and have advantages in tourism investment and advanced technology, and thus, they lead the sustainable tourism development model. With its developed economy, the eastern region has higher tourism eco-efficiency than the economically backward western region. Tourism development in the western region is difficult because of the fragile ecological environment and the constraints related to economy, technology, and transportation, resulting in the low tourism eco-efficiency.

With the proposal of “Low-carbon Green Tourism” and the promulgation of “Opinions on Escalating the Tourism Development,” “Outline for National Ecotourism Development,” and “Several Suggestions on Promoting the Tourism Reform and Development,” tourism economy has transitioned into a novel normal phase. The structural reform on the supply side was effectuated for the purpose of industrial structure optimization, and by 2016, immense improvement was achieved in terms of the situation of tourism ecological security [57]. Particularly, in 2019, the Yellow River Basin ecological protection and high-quality development strategy evolved into a national strategy. The focus of the tourism industry has transformed from scale expansion to quality improvement. The low-carbon and intensive tourism development model and the establishment of tourism ecological compensation mechanism have enhanced the tourism eco-efficiency. Additionally, the proportion of the high- and higher-efficiency areas has increased, whereas the proportion of the low- and lower-efficiency areas has decreased (Fig 4). The development of tourism eco-efficiency presents a fluctuating upward trend (Fig 3). However, the development trend of tourism eco-efficiency has been satisfactory [56].

Differences were observed in the development trends of different areas, with high- and low-efficiency areas concentrated in the middle and upper reaches, revealing a gradually decreasing spatial pattern of “middle reaches > lower reaches > upper reaches.” This result conforms to those of the study by Cheng and You [56], but different from those of Zhang, Duan, Wang, Han and Wang [58], which can be attributed to the differences in the regional energy consumption structure and carbon emission estimation methods [59], as well as the differences in undesirable output results. The middle and lower reaches of the Yellow River Basin constitute a large weight in terms of economic aggregate, population, environment, and other aspects. The fluctuation trend of efficiency values in the middle and lower reaches of the Yellow River Basin was close to the overall trend of the Yellow River Basin. We discovered that the value of tourism eco-efficiency in the river basin was stable(Fig 2I), and the regional gap gradually narrowed. Tourism is a vital economic growth point in the economically backward areas, which have a latecomer advantage compared with the economically developed areas [60]. Apart from the immense tourism eco-efficiency upgrading in the upstream area, the gap among the Basin’s upper, middle, and lower reaches also continues to decrease.

### Evolution of spatial pattern of tourism eco-efficiency

Tourism eco-efficiency of the Yellow River Basin is typified by spatial agglomeration and interdependence(Table 2), and its spatial pattern has changed over the years. The spatial distributions of cold and hot spots are mutually validated with the local spatial interrelation (Fig 7). The spatial agglomeration characteristics of tourism eco-efficiency in the Yellow River Basin are obvious, and they are influenced by the spatial spillover effect of tourism eco-efficiency [36, 61]. The agglomeration areas in the middle reaches are distributed in Shanxi Province and those in the upper reaches are distributed in Ningxia, Inner Mongolia, Gansu, and Qinghai provinces. Under cyclic accumulation and path dependence, Shanxi Province has formed resource-intensive industries majorly dependent on coal. Although these industries have promoted the economic development of cities, they have also led to environmental pollution. With the high per capita income, the desire of residents to pursue a better life is gradually increasing, resulting in the transformation of the regional green development model [41]. Furthermore, the rich and unique natural and cultural landscape in Shanxi is conducive to the development of ecotourism. The government and enterprises in the province have increased the investment in clean technology, which can thus reduce energy consumption and pollutant emission and improve the tourism eco-efficiency rapidly. Therefore, the province has become a high- and higher-efficiency-concentration area and hot spot area of tourism eco-efficiency in the Yellow River Basin as well as a center of gravity distribution area. The upstream region is a low and low efficiency concentration area and cold spot area (Figs 6 and 7), located in western China (Fig 1) and influenced by natural factors including climate and terrain. Therefore, its ecological environment is fragile. Furthermore, the region is far from the coastal areas, and the levels of economic development, science and technology development, and transportation in this region are low. Although the region has abundant cultural tourism resources, developing them is difficult, which results in the low tourism eco-efficiency. During the period from 2009 to 2019, the trend of the standard deviation ellipse of efficiency value was north by east—south by west, with no obvious change in the trajectory of the average center point (Fig 8), suggesting that the spatial pattern of tourism eco-efficiency did not change considerably during the period. Relying on resource endowment, geographical advantages, and transportation and economic conditions, the eastern region of the Yellow River Basin results in the development of tourism economy, forming an extensive development model that achieves economic benefits by increasing the input and expanding scale. Ignoring ecological benefits has resulted in a shift of focus from northeast to southwest. In 2014, the elliptical area was the smallest and its flatness was the largest, with no significant changes in other years (Fig 9). This result proves that the tourism eco-efficiency value changed considerably in 2014, and the change of China’s tourism policy and tourism conversion efficiency from 2009 to 2019 was the abrupt point of tourism eco-efficiency [44].

### Analysis of the influencing factors of tourism eco-efficiency

The enhancement and model transformation of tourism eco-efficiency in the Yellow River Basin are influenced by human, social, and economic factors. Economic development level, market opening degree, and tourism industry agglomeration intensity considerably influence the efficiency value (Fig 11). The level of economic progress not only provided financial support for the optimization of the regional infrastructure and the introduction of advanced technology but also increased residents’ income and enhanced their purchasing desire and consumption power. Market opening is a major contributor to carbon emission [62]. Although stakeholders’ ideas and investment models in tourism activities have changed considerably, they actively participated in market competition, injected new vitality into the tourism market, and improved the willingness of tourists to pay [63]. However, tourist arrivals increase per capita carbon emissions[64]. Large-scale tourist activities further increase carbon emission [65]. This influx introduces high-energy consuming and heavily polluting enterprises, which results in the “pollution paradise” effect [66], increasing the local ecological environmental pressure and hindering the enhancement of tourism eco-efficiency. In addition, tourism industry agglomeration positively affects tourism eco-efficiency in the Yellow River Basin, which indicates that industrial agglomeration forms technology spillover, learning and competition effects, and promotes the optimal allocation of tourism factors. Guided by green development, the Yellow River Basin has constantly optimized its industrial structure, and the tourism development model has gradually changed from scale expansion to quality and benefits. Science and technology can not only reduce pollution emissions but also provide technical support for tourism pollution control. Limited by the economic development level in the upper and middle reaches, the contribution of science and technology input to tourism eco-efficiency in these reaches is less than that in the downstream area. The upstream area is large and sparsely populated, with weak infrastructure and low tourist attraction. Therefore, the effect of pollution prevention and control is obvious in the region, with a strong driving force of environmental regulation. The middle and downstream areas comprise resource-intensive cities with prominent environmental problems. However, environmental regulations have increased the production costs of tourism enterprises and reduced economic benefits, indicating that the driving force is weak. The level of traffic indicates the possibility of tourists entering the area, and the more developed the traffic, the greater the accessibility of tourist. The upstream area is large and sparsely populated, with backward infrastructure and inconvenient transportation. Increasing the density of the road network can improve its accessibility and tourism eco-efficiency. The downstream area is densely populated, with flat terrain, and the increase in the road network density can increase people’s willingness to travel. The middle reaches were in the Loess Plateau, with a fragile ecological environment and the road network density reaching a certain threshold, which may not be conducive to the tourism eco-efficiency. Therefore, the traffic level drives the enhancement of upstream and downstream tourism eco-efficiency but inhibits the improvement of midstream tourism eco-efficiency.

## Conclusion

In this study, 63 cities in the Yellow River Basin were included to construct an evaluation index system of tourism eco-efficiency in the basin. The trend surface analysis, spatial autocorrelation analysis, cold-hot spot analysis, and elliptic standard deviation analysis were comprehensively adopted for quantitatively analyzing the spatiotemporal distribution characteristics of tourism eco-efficiency from 2009 to 2019. The GTWR model was adopted for exploring the spatiotemporal influential factors of tourism eco-efficiency value in the Yellow River Basin, which can provide a scientific decision-making basis for the implementation of high-quality development strategies in the Yellow River Basin. The conclusions can be summarized as follows:

1. The level of tourism eco-efficiency in the Yellow River Basin has not been high, exhibiting a fluctuating upward trend. The trend exhibits an “oscillation period” and an “improvement period,” and the spatial distribution remains uneven, indicating a gradually decreasing spatial pattern of “middle reaches > lower reaches > upper reaches.”
2. Tourism eco-efficiency in the Yellow River Basin exhibits significant spatial interdependence and agglomeration characteristics, and the spatial pattern has not changed considerably over the years. The high- and higher-efficiency-concentration areas and hot spot areas of tourism eco-efficiency are distributed in the middle reaches of Shanxi Province, whereas the low and low concentration areas and cold spot areas are distributed in the upper reaches, and their barycenter tracks move from northeast to southwest.
3. Tourism eco-efficiency in the Yellow River Basin has been influenced by various factors, and the effect of each factor on the efficiency value differs significantly. The order of influence degree is economic development > market opening > tourism industry cluster > environmental regulation > science and technology > traffic.

However, the present study has some shortcomings. First, because of the difficulty of data acquisition, this study considered the tertiary industry employees to represent the tourism labor force and the converted carbon emission as the non-expected output. Second, this study revealed that tourism eco-efficiency in the Yellow River Basin shows a significant spatial interdependence relationship, and the local convergence characteristics are significant; however, these results could not be discussed because of space limitations. Future studies should focus on improving the evaluation index system of tourism eco-efficiency and obtaining relevant data on the tourism industry of prefecture-level cities in the Yellow River Basin through field research. Moreover, the spatial correlation characteristics of tourism eco-efficiency in the Yellow River Basin should be studied and the internal driving mechanism of the spatial correlation should be explored.

## Supporting information

S1 Data (XLSX)

S2 Data (XLSX)
